# Integrating Hepatitis C Care for Opioid Substitution Treatment Patients Attending General Practice: Feasibility, Clinical, and Cost-Effectiveness Analysis

**DOI:** 10.2196/35300

**Published:** 2022-08-23

**Authors:** Geoff McCombe, Davina Swan, John S Lambert, Eileen O’Connor, Zoe Ward, Peter Vickerman, Gordana Avramovic, Des Crowley, Willard Tinago, Nyashadzaishe Mafirakureva, Walter Cullen

**Affiliations:** 1 School of Medicine University College Dublin Dublin Ireland; 2 Mater Misericordiae University Hospital Dublin Ireland; 3 Bristol Population Health Science Institute Bristol Medical School University of Bristol Bristol United Kingdom; 4 Health Service Executive Dublin Ireland

**Keywords:** hepatitis C, integrated HCV care, people who inject drugs, primary health care

## Abstract

**Background:**

Hepatitis C virus (HCV) infection is common among people who inject drugs, yet well-described barriers mean that only a minority have accessed HCV treatment. Recent developments in HCV diagnosis and treatment facilitate innovative approaches to HCV care that improve access to, and uptake of, care by people who inject drugs.

**Objective:**

This study aims to examine feasibility, acceptability, likely clinical effectiveness, and cost-effectiveness of an integrated model of HCV care for patients receiving opioid substitution treatment in general practice.

**Methods:**

A pre- and postintervention design with an embedded economic analysis was used to establish the feasibility, acceptability, and clinical and cost-effectiveness of a complex intervention to optimize HCV identification and linkage to HCV treatment among patients prescribed methadone in primary care. The “complex intervention” comprised general practitioner (GP)/practice staff education, nurse-led clinical support, and enhanced community-based HCV assessment of patients. General practices in North Dublin were recruited from the professional networks of the research team and from GPs who attended educational sessions.

**Results:**

A total of 135 patients from 14 practices participated. Follow-up data were collected 6 months after intervention from 131 (97.0%) patients. With regard to likely clinical effectiveness, among patients with HCV antibody positivity, there was a significant increase in the proportions of patients who had a liver FibroScan (17/101, 16.8% vs 52/100, 52.0%; *P*<.001), had attended hepatology/infectious diseases services (51/101, 50.5% vs 61/100 61.0%; *P*=.002), and initiated treatment (20/101, 19.8% vs 30/100, 30.0%; *P*=.004). The mean incremental cost-effectiveness ratio of the intervention was €13,255 (US $13,965.14) per quality-adjusted life-year gained at current full drug list price (€39,729 [US $41,857.48] per course), which would be cost saving if these costs are reduced by 88%.

**Conclusions:**

The complex intervention involving clinical support, access to assessment, and practitioner education has the potential to enhance patient care, improving access to assessment and treatment in a cost-effective manner.

## Introduction

Hepatitis C virus (HCV) infection is an important issue for general practice—it is a common infection, often not diagnosed or treated and associated with potentially preventable chronic liver disease [1]. It is estimated that 10 million people who inject drugs globally and 0.7 million people who inject drugs in Europe have been infected with HCV [2]. Despite the high prevalence among people who inject drugs, many are unaware of their infection and few have received treatment for the infection. In Europe, estimates of undiagnosed infection among people who inject drugs range from 24% to 76% [3], while among people who inject drugs diagnosed with chronic HCV, 1%-19% have commenced HCV treatment [3].

In Ireland, 20,000-30,000 people are chronically infected with HCV [4], with injecting drug use the primary risk factor in 80% of cases [5]. Methadone is the only form of opioid substitution treatment (OST) prescribed in Ireland and is provided by addiction treatment centers, specialized general practitioners (GPs), and in prisons, for the treatment of opioid use disorder/opioid dependence [6]. A previous study in Dublin found that 77% (151/196) of patients on OST in general practices had been screened for HCV, and of those who were HCV antibody positive, just 35% (36/104) had received follow-up HCV-RNA testing, 30% (31/104) had been referred to a hepatology clinic, and 3% (3/104) had initiated HCV treatment [7]. Several barriers impede or discourage people who inject drugs from accessing HCV testing, evaluation, and treatment. These include stigma, restrictions around HCV treatment eligibility, not being referred for treatment, fear of HCV investigations (eg, liver biopsy)/treatment side effects, and competing priorities for people who inject drugs [8-11].

The World Health Organization (WHO) has developed a Global Health Sector Strategy (GHSS) to eliminate viral hepatitis as a public health threat by 2030 [12]. Increasing prevention, diagnosis, and treatment is a priority, especially among people who inject drugs. As many people who inject drugs are unaware of their infection, new strategies to reach such individuals are necessary, including testing strategies to increase recognition of HCV and improved care pathways to ensure those diagnosed are successfully linked to HCV evaluation and treatment.

Historically, specialist physicians have provided HCV treatment, usually from tertiary hospital outpatient clinics [1]. However, recent developments in HCV diagnosis and treatment facilitate innovative approaches to community-based HCV care, whereby a patient’s treatment pathway can start in community-based clinics and general practices, resulting in improved access to and uptake of care by people who inject drugs. These include point-of-care tests for HCV (including dried blood spot and saliva testing) [13] and transient elastography (FibroScan), which allow specialist evaluation and noninvasive staging of HCV-related liver disease in a community setting [14]. Several European studies have reported on the feasibility of FibroScanning as a screening tool for people who inject drugs, with high rates of acceptance and uptake within various treatment and street outreach settings [14,15]. Furthermore, direct-acting antivirals (DAAs) are taken orally and for shorter periods, associated with fewer side effects, and are therefore more likely to be better tolerated. Cure rates of over 90% have been reported among people who inject drugs [16,17]. In Ireland, DAAs are currently the standard of care for HCV treatment, which is generally provided in specialist hospital services.

Unrestricted access to DAAs and substantial scale-up of treatment are necessary to achieve WHO 2030 targets [18], and engaging people who inject drugs in the continuum of HCV care from testing through to treatment is key to this [18]. The establishment of culturally appropriate and flexible models of care that meet their specific needs and are adapted to the circumstances of people who inject drugs will be essential to optimize HCV diagnosis and linkage to HCV evaluation and treatment among people who inject drugs [18,19].

In Ireland and many European Union countries, primary care is increasingly providing continuing care for people who inject drugs and Irish general practice has been a leader in the introduction and expansion of harm-reduction services, including opioid substitution OST, needle and syringe programs, and naloxone provision. These services have been effective in engaging opiate users in treatment, reducing HIV and HCV transmission, and reducing-drug related morbidities [20]. General practice is therefore an appropriate setting to deliver enhanced HCV care to patients being prescribed methadone. Practitioner education and nurse liaison support can increase rates of HCV screening and referral to specialist HCV care in this setting [21].

The “HepLink” study aimed to develop, implement, and evaluate a complex intervention to integrate primary care and specialist HCV care to enhance HCV identification, evaluation, and treatment among patients being prescribed methadone. While there is no sharp boundary between complex and simple interventions, complex interventions are described as interventions that contain several interacting components [22]. As such, the “HepLink” complex intervention involving practitioner education and HCV nurse outreach/clinical support to primary care was developed and implemented in general practices providing methadone treatment. This paper aims to evaluate the feasibility and acceptability of this intervention to primary care providers and patients, and to determine its likely clinical effectiveness and cost-effectiveness.

## Methods

### Study Design

A pre- and postintervention design with an embedded economic analysis was used to establish the feasibility, acceptability, and clinical and cost-effectiveness of a complex intervention to optimize HCV identification and linkage to HCV treatment among patients on OST in primary care in North Dublin [23]. A sample of 24 OST-prescribing GP practices were invited to participate in the study from the professional networks of members of the research consortium and from those GPs who attended a series of educational masterclasses on “Advances in Hepatitis C Treatment in the Community.” This masterclass symposium series highlighted the benefits of educational seminars as a way of delivering current best practice in the treatment of HCV infection to a multidisciplinary audience and a useful vehicle for recruiting general practices to the study [24].

### Setting

In Ireland, currently there are 2 types of settings in which OST is delivered in the community: specialist addiction clinics and general practice. All patients receiving OST are registered on the Central Treatment List. “Level 1” GPs are responsible for the treatment of stabilized opiate-dependent persons [25] referred from specialist addiction clinics or from “Level 2” GPs. Practice as a “Level 1” GP requires completion of a recognized training program delivered by the Irish College of General Practitioners (ICGP) and regular educational updates. The GP is audited by the ICGP/Health Services Executive (HSE) Audit Committee. “Level 1” GPs can treat up to a maximum of 15 patients. A “Level 2” GP has undergone additional training, can initiate OST, and prescribe for a greater number of patients (up to a maximum of 35 patients or a maximum of 50 in a partnership with 2 or more doctors in their own practice [26]). As of August 31, 2016, there were 9652 patients receiving treatment for opiate use in Ireland (excluding patients in prisons), which included 4150 patients being treated by 350 GPs in the community [27].

The National Hepatitis C Treatment Programme oversees access to DAA treatment and provides HCV screening guidelines to identify risk groups for screening [28]. Prior to 2017, access was organized according to clinical need and restricted to those who were infected with HCV through blood and blood products and those scoring over 8.5 kPa on FibroScan. In early 2017 the criteria were revised to remove this threshold, but a limited health care budget and the high cost of DAAs continue to restrict the numbers who can avail of treatment.

### Participants

A total of 14 general practices consented to participate in the study and were asked to recruit 10 consecutive patients on OST (ie, methadone), aged at least 18 years, and who were attending the practice for any reason during the recruitment period. Based on the recommendations for good practice in feasibility studies [29], and our previous feasibility studies among people who inject drugs [30,31], we estimated that 140 patients (attending 14 general practices) would be adequate to estimate recruitment and retention rates (ie, feasibility) and provide data on acceptability of the intervention, to inform a future definitive intervention (trial). This sample size exceeded that recommended for feasibility studies of between 24 and 50 and allowed feasibility assessments within both Level 1 and Level 2 practices [29]. A detailed description of recruitment procedures has been reported separately [32].

### Intervention

**Figure 1:**
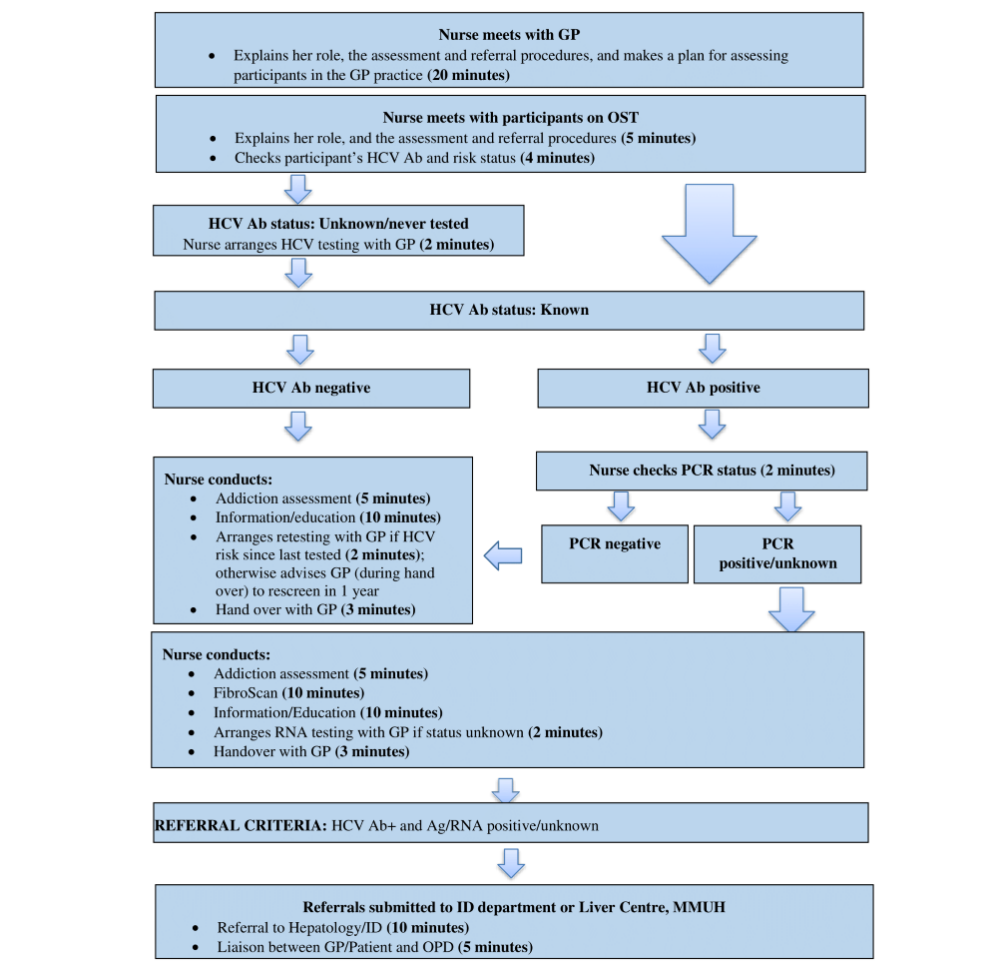
Flowchart describing nurse-led clinical assessment. Ab: antibody; Ag: antigen; GP: general practitioner; HCV: hepatitis C virus; MMUH: Mater Misericordiae University Hospital; OST: opioid substitution treatment; PCR: polymerase chain reaction.

The complex intervention was delivered to the 14 primary care sites and consisted of outreach by a specialist HCV nurse into primary care sites to optimize interaction and integration between primary and secondary care. Informed by the HepCare Europe Education Masterclass series [24], the nurse provided HCV education, clinical support, and community-based HCV evaluation of patients. The protocol for clinical assessment by the nurse is described in Figure 1.

### Data Collection

Clinical records of participating patients were reviewed prior to the implementation of the complex intervention and 6 months after the intervention by a member of the research team (EO’C) and data were extracted on HCV care processes and outcomes. We measured feasibility as the number of practices (14/24) and patients (135/140) who were recruited to the study (recruitment rate) and those on whom follow-up procedures were completed (retention rate). Acceptability of the complex intervention was assessed as uptake of its component interventions by both GPs and patients, which included (1) practitioner education; (2) HCV nurse outreach/clinical support; (3) community-based HCV evaluation of patients, including mobile elastography.

### Process/Outcome Measures

In addition to demographic characteristics, the following data on the HCV cascade of care (between diagnosis and sustained virologic response [SVR]) were extracted for each patient from their clinical record immediately prior to the implementation of the intervention and 6 months after the intervention.

### Blood-Borne Virus Care

The following were considered: HCV antibody testing and status; HCV RNA and antigen (Ag) testing and status; whether the patient had been referred to a hepatology or infectious diseases specialist; had attended a hepatology or infectious diseases specialist; had been assessed by FibroScan; FibroScan score (kPa); had initiated HCV treatment; had completed HCV treatment; and achieved SVR, which means that the HCV is not detected in the blood 12 weeks or more after completing DAA treatment [33].

### Data Analysis

Feasibility and acceptability measures were summarized with counts (percentages) for categorical variables and median (IQR) for continuous variables. Care process and outcome measures were analyzed using intention-to-treat principles. While the study was not powered to assess effectiveness, the possible impact of the intervention on care process measures was measured by examining the proportion of participants before and 6 months after the intervention who had received HCV testing, and the proportion of HCV antibody–positive patients before and 6 months after the intervention who had ever received follow-up Ag or RNA testing, been referred to a hepatology/infectious diseases service, attended a hepatology/infectious diseases service, been FibroScanned, initiated HCV treatment, and completed HCV treatment. Possible impact of the intervention on care outcomes was measured by examining the proportion of those screened who tested HCV antibody positive and the proportion of patients with HCV antibody positivity achieving SVR before and 6 months after the intervention. Paired binary differences before and 6 months after the intervention for selected process measures were compared using the McNemar test, with *P* values <.05 considered statistically significant. All analyses were done using Stata 15 (StataCorp).

### Cost-Effectiveness Analysis

A Markov model of HCV disease progression and treatment was used to estimate the impact and cost-effectiveness of the HepLink intervention compared with the current standard of care pathway of antibody testing and referral by primary care practitioners. The model was used to track disease progression for anyone with chronic HCV and the effect of treatment in reducing levels of liver disease (details in the Supplementary Material entitled “HepLink Cost-Effectiveness Analysis” in Multimedia Appendix 1 [28,34-49]). Health benefits were measured in quality-adjusted life-years (QALYs). Pre-HepLink data suggested that 6% (95% CI 1%-12%) of diagnosed chronically infected patients on OST are treated per year at baseline, which was used as the background treatment rate in both the baseline and intervention scenarios.

HepLink intervention data were used to parameterize the initial fibrosis staging of the intervention cohort and provide subsequent intervention outcomes in terms of proportion of individuals linked to care and treated. Primary cost data for the HepLink intervention (including costs for staff, diagnostics, room rental, overheads, and training) and subsequent HCV treatment were collected through interviews (in 2017 euros) with intervention staff and from financial records. Other model parameters such as disease transition rates, death rates, health utilities, and health care costs for different HCV disease stages, sustained viral response cure rates for treatment, and disease progression rates after SVR came from the existing literature [34-36,50] (see Supplementary Tables 1 and 2 in Multimedia Appendix 1).

The cost-effectiveness analysis was undertaken for full (€39,729 [US $41,857.48] per course) and 10% of HCV drug list price over a 50-year time horizon with a 5% discount rate [37]. The incremental cost-effectiveness ratio (ICER) in terms of the incremental cost per incremental QALYs gained of the intervention was used to determine the cost-effectiveness at Ireland’s willingness-to-pay (WTP) threshold (€30,000 [US $31,607.25] per QALY [37]). We used probabilistic sensitivity analysis (PSA) to determine the effect of parameter uncertainty (distributions given in see Supplementary Tables 1-3 in Multimedia Appendix 1) on the cost-effectiveness projections, and also undertook univariate sensitivity analyses to assess the effect of some of the model and intervention assumptions. This included the effect of the background treatment rate and HCV drug list price on the mean ICER.

### Ethics Approval

The study has been approved by the Mater Misericordiae University Hospital Research Ethics Committee (Ref: 1/378/1722)

## Results

### Feasibility

**Figure 2:**
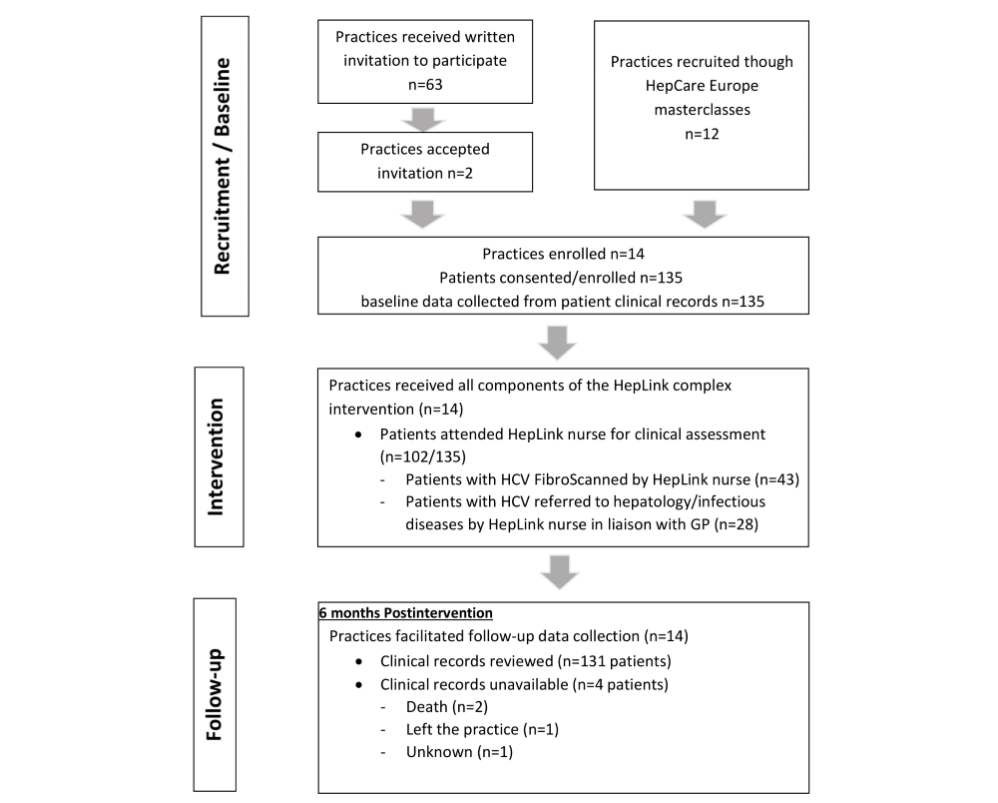
Study flow diagram. GP: general practitioner; HCV: hepatitis C virus.

Fourteen practices participated in the study out of 24 practices that were invited, and the 14 practices recruited and obtained consent from 135 patients out of 140 who were invited to participate (Figure 2). All 14 practices facilitated follow-up data collection 6 months after the intervention and follow-up data were collected from the clinical records of 131 (97.0%) patients; clinical records of 4 patients were unavailable at follow-up for the following reasons: the patients were deceased (n=2), had left the practice (n=1), or unknown reason (n=1). As many as 11 (8.4%) of the 131 patients on whom follow-up data were collected had incomplete clinical records for the follow-up period for the following reasons: during the follow-up period they transferred to another GP/addiction service (n=4), were incarcerated (n=2), left the practice (n=1), no longer on OST (n=1), or unknown reasons (n=3).

### Baseline Characteristics of Participants

Of the 14 practices enrolled in the study, 7 were Level 1 prescribers (n=53 patients) and 7 were Level 2 prescribers (n=82 patients). Baseline characteristics of 14 practices and the 135 participating patients are outlined in Tables 1 and 2, respectively, and previously reported by Murtagh et al [32].

**Table 1:** Baseline characteristics of practices (n=14).

Characteristic			Value, n (%)
**Level of training in providing addiction-related care**			
	Level 1 general practitioner	7 (50)	
	Level 2 general practitioner	7 (50)	
**Sex of general practitioner**			
	Male	10 (71)	
	Female	4 (29)	
**Practice nurse**			
	Yes	14 (100)	
	No	0 (0)	

**Table 2:** Baseline characteristics of the patients (n=135).

Characteristic		Value
**Sex, n (%)**		
	Male	97 (71.9)
	Female	38 (28.1)
Age (years), median (IQR)		42 (38-48)
On opioid substitution treatment, n (%)		135 (100)

### Acceptability Measures

Acceptability of the complex intervention was assessed as uptake of its component interventions. The measures included (1) practitioner education, (2) HCV nurse outreach/clinical support, (3) community-based HCV evaluation of patients, including FibroScan. All 14 primary care sites received practitioner HCV education and HCV nurse outreach/clinical support. Community-based HCV evaluation of patients was conducted at all practices, with 102/135 (75.6%) participating patients attending the HCV nurse for an on-site clinical assessment. The clinical protocol involved FibroScanning patients with chronic infection and those with HCV antibody positivity whose RNA/Ag status was unknown. As many as 43 (75%) of the 57 patients who were HCV antibody positive and RNA/Ag positive or unknown were FibroScanned by the HCV nurse. Of the remainder, 5/14 (36%) patients had recently been FibroScanned at the hospital and therefore were not FibroScanned again, 4/57 (7%) patients declined to be scanned, a valid FibroScan reading was unable to be obtained for a further 4/57 (7%) patients, and 1 patient’s (2%) FibroScan was deferred until RNA/Ag testing had been conducted by their GP. The median (IQR) liver stiffness score for the 43 patients FibroScanned by the HCV nurse was 7.5 (5.7-13.8) kPa. As many as 19 (44%) of the 43 patients FibroScanned scored over 8.5 kPa and 12/43 (28%) had cirrhosis (scored >12.5 kPa).

### Clinical Effectiveness

The proportion of patients tested for HCV did not significantly increase 6 months after the intervention compared with the preintervention screening (128/135, 94.8% vs 128/131, 97.7%; *P*=.25; Table 3). Among those screened for HCV, compared with the preintervention status, there were no significant changes in the proportion with HCV antibody–positive test at 6 months after the intervention (100/128, 78.1% vs 99/128, 77.3%; *P=*.99).

**Figure 3:**
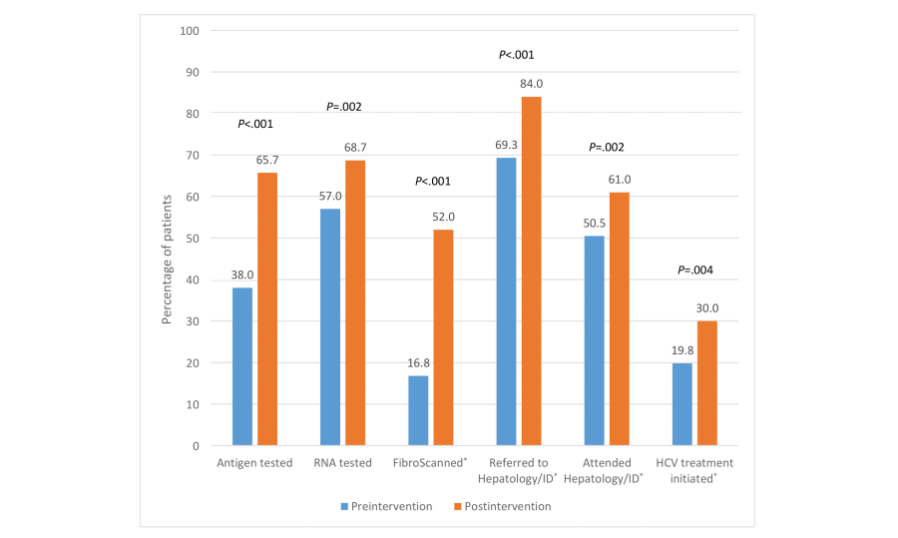
Proportion of patients with HCV antibody positivity receiving each step in the cascade of HCV care before the intervention and 6 months after the intervention. HCV: hepatitis C virus; ID: infectious disease. *Includes 1 patient who was HCV antibody negative but antigen positive.

Significant improvements were observed across all steps in the care cascade at 6 months after the intervention (Figure 3). One participant was HCV Ag positive and antibody negative before the intervention and 6 months after the intervention and was included in the analysis of subsequent steps in the care cascade at both time points. Among patients who were Ag/RNA positive or whose RNA/Ag status was unknown, the proportion who had been referred to a hepatology or infectious diseases service was significantly higher 6 months after the intervention (70/101, 69.3% vs 84/100, 84.0%; *P*<.001), as was attendance at a hepatology or infectious diseases service (51/101, 50.5% vs 61/100, 61.0%; *P*=.002). There was a 35% significant increase in the proportion of patients with HCV antibody positivity or Ag/RNA positivity/unknown status who had been FibroScanned (17/101, 16.8% vs 52/100, 52.0%; *P*<.001).

The proportion of patients with Ag/RNA positivity who had started HCV treatment was significantly higher, with 10 additional patients initiating treatment (20/101, 19.8% vs 30/100, 30.0%; *P*=.004). As many as 14 (13.9%) of the 101 patients with HCV Ag/RNA positivity had completed HCV treatment before the intervention and 21/100 (21.0%) had completed HCV treatment 6 months after the intervention (*P*=.16). The proportion of patients with HCV Ag/RNA positivity who had achieved SVR was 14/101 (13.9%) before the intervention and 19/100 (19.0%) 6 months after the intervention (*P*=.16).

**Table 3:** Baseline/6-month follow-up data.

Data		Baseline	Follow-up	*P* value^a^
Patients on whom data were collected, n		135	131	
**HCV^b^ testing, n**		135	131	
	HCV antibody test, n/N (%)	128/135 (94.8)	128/131 (97.7)	.25
	HCV antibody positive, n/N (%)	100/128 (78.1)	99/128 (77.3)	>.99
**Among patients with HCV antibody positivity, n/N (%)**				
	HCV antigen test	38/100 (38.0)	65/99 (65.7)	<.001
	HCV antigen positive	22/38 (57.9)	37/65 (56.9)	
	HCV RNA test	57/100 (57.0)	68/99 (68.7)	.002
	HCV RNA positive	35/57 (61.4)	37/68 (54.4)	.63
**Management of patients with HCV antibody positivity**				
	FibroScanned, n/N (%)	17/101 (16.8)	52/100 (52.0)	<.001
	FibroScan score (kPa)—lifetime, median (IQR)	6.4 (5.6-8.4)	7.4 (5.5-10.9)	
	Referral to hepatology or infectious diseases, n/N (%)	70/101 (69.3)	84/100 (84.0)	<.001
	Attended hepatology or infectious diseases services, n/N (%)	51/101 (50.5)	61/100 (61.0)	.002
**Treatment of patients with HCV antibody positivity, n/N (%)**				
	HCV treatment initiated	20/101 (19.8)	30/100 (30.0)	.004
	HCV treatment completed	14/101 (13.9)	21/100 (21.0)	.16
	SVR^c^ attained	14/101 (13.9)	19/100 (19.0)	.32

^a^*P* values represent significance levels of the McNemar test.

^b^HCV: hepatitis C virus.

^c^SVR: sustained virologic response.

### Cost-Effectiveness Analysis

Direct costs of the intervention were €85,439 (US $90,016.39) over the 15-month intervention period, with the treatment costs increasing by €223,112 (US $235,065.23; at full treatment cost). The main components of the intervention costs were €59,198 (US $62,369.53) for set up and implementation and €26,241 (US $27,646.86) for the nurse liaison component. Over the intervention, 43/57 (75%) individuals with antibody positivity with positive or unknown RNA/Ag status were FibroScanned by the nurse; 28/57 (49%) of these were referred and 10/28 (36%) started treatment in secondary care. The cost per person FibroScanned was €1507 (US $1587.74; setup costs annualized over 5 years and 43 FibroScanned patients). Model projections suggest savings of €113,769 (US $119,864.17) in HCV-related care and 15 QALYs saved among the 10 additional treated individuals over 50 years. This gives the incremental cost of HepLink as €194,782 (US $205,217.45; direct intervention costs plus treatment costs minus HCV-related care costs saved). At full drug costs, our projections suggest HepLink was cost-effective with a mean ICER of €13,255 (US $13,965.14) per QALY saved and 98% of PSA runs being below the WTP threshold (€30,000 [US $31,607.25] per QALY; see Supplementary Figure 2 and Supplementary Table 3 in Multimedia Appendix 1). Together, uncertainty in the progression rates from Metavir stages F3 and F4 accounted for most of the variation in the ICER (36% and 40%, respectively). The intervention becomes cost saving at 12% of the full drug costs, with all of the PSA runs being below the WTP threshold for Ireland and 48% being cost saving (see Supplementary Figure 3 in Multimedia Appendix 1).

## Discussion

### Principal Findings

A complex intervention (practitioner education, practice-based assessment, and nurse liaison) may enhance HCV care among patients being prescribed methadone in primary care and is likely to be feasible, acceptable, effective, and cost-effective, with care enhanced specifically for those patients who are HCV positive. By utilizing a liaison nurse to provide HCV education, clinical support, and evaluation of patients, the “HepLink” intervention helps overcome barriers such as patients not being referred for treatment and also patients’ fear of treatment, and provides a more flexible and accessible model of HCV care.

### Strengths and Limitations

The key findings from this study provide a better understanding of how to overcome barriers and improve access to care, which can inform policy and service development, and contribute to health both locally and internationally. This study has made an important impact on patient care and supported GPs in making important decisions on HCV testing and onward referral. The strengths of the study are the large numbers who were followed up at 6 months and the uniqueness of the population (OST patients in primary care), which are rarely reported in the literature. The intervention is scalable, and its initial success suggests that it could potentially be implemented elsewhere and used to guide service development and policy internationally.

However, there were limitations to the study design. First, the study used a nonprobability sampling strategy. Although this results in a lower generalizability of findings and inability to calculate CIs or margin of error, we felt it was an appropriate sampling strategy to use for a population consisting of OST patients in primary care, especially when conducting a feasibility study with lower sample sizes that makes probability sampling impractical [51]. Second, there was no control group that prevents the analysis of any preexisting trends or accounting for the possibility that other factors occurred at the same time as the intervention. However, the findings from this feasibility study can inform power calculations for a future large-scale randomized control trial using the “HepLink” complex intervention. The third limitation is that there may be a lack of generalizability to those not in addiction treatment and potential bias may occur from GPs who are more motivated and enthusiastic about the issue under study being overrepresented among those recruited. Because of their interest in the issue, self-selected GPs in the study may be providing better HCV care to their patients than the wider GP population. However, the profile of GPs and patients participating in the study was similar to other Irish studies despite this potential bias [7,52].

### Comparison With Prior Work

Our findings, compared with previous studies conducted in Ireland [7,8,21,53], indicate higher attendance and treatment rates than previously reported (Figure 3). In our baseline data these increases reflect the general increase in HCV outreach programs and better education of GPs in Ireland since the introduction of DAAs. However, further increases in our postintervention data indicate the likely effectiveness of the HepCare (HepLink) intervention in enhancing access to specialist assessment and HCV treatment, and better education of GPs in HCV care.

The most significant increase was observed in FibroScan rates: from 16.8% (17/101) before the intervention to 52.0% (52/100) after the intervention. This study also saw an increase in those initiating treatment: 19.8% (20/101) before the intervention versus 30.0% (30/100) after the intervention. This increase is lower than the 31% increase achieved during the HepCATT study intervention year [54]. However, the higher increase achieved in the HepCATT study can be accounted for by the longer intervention period (1 year); the placement of a half-time nurse facilitator to address diagnosis, assessment, and integration within the HCV cascade of care at each clinic undergoing the intervention; and the establishment of local peer champions to support patients.

The success of this intervention in a primary care setting underlines the results of Project ECHO, which suggests HCV care delivered through primary care can be as safe and effective as that provided by specialists at an acute medical center [55]. The ETHOS study suggests that the highly marginalized population of people who inject drugs can achieve a similar adherence to treatment as other populations, with 74% completing their intended duration of treatment [5]. This was also reflected in the results of this study with significant improvements observed across all steps in the care cascade at 6 months after the intervention. The proportions of patients who had a liver FibroScan, had attended hepatology/infectious diseases services, and initiated treatment were significantly higher 6 months after the intervention. More recently, the role of primary care advanced practice nurses in engaging patients with treatment has been highlighted [56].

### Future Directions

The findings from this feasibility study can inform the design of a future large-scale randomized control trial using the “HepLink” complex intervention. Furthermore, incorporating a peer support model to aid and improve access and adherence to the HCV care pathway for the most vulnerable patients could enhance treatment completion rates and SVR outcomes.

### Conclusions

The population studied was exposed to the HepLink intervention and thus this study supports further development and broader implementation of the intervention. The HepLink intervention has the potential to impact on patient care, improving access to care and providing quality health care to marginalized populations who might otherwise remain untreated. The data collected enhance the scientific understanding of interventions that contribute to health and social gain and can inform national policy and service development. The authors are actively engaged with key stakeholders and policymakers to ensure that the HepLink project contributes to policy and practice.

## Appendix

Multimedia Appendix 1 HepLink Cost-effectiveness Analysis Supplementary Material.

## Abbreviations

Ag: antigen
DAA: direct-acting antiviral
GHSS: Global Health Sector Strategy
GP: general practitioner
HCV: hepatitis C virus
HSE: Health Services Executive
ICER: incremental cost-effectiveness ratio
ICGP: Irish College of General Practitioners
ID: infectious disease
OST: opioid substitution treatment
PSA: probabilistic sensitivity analysis
QALY: quality-adjusted life-year
SVR: sustained virologic response
WHO: World Health Organization
WTP: willingness-to-pay
