# Supplementary material for: Integrating Hepatitis C Care for Opioid Substitution Treatment Patients Attending General Practice: Feasibility, Clinical, and Cost-Effectiveness Analysis
Source: Interact J Med Res. 2022 Aug 23;11(2):e35300. doi: 10.2196/35300 (PMC9449831; doi:10.2196/35300)

HepLink Cost-effectiveness Analysis Supplementary Material

Background

In Ireland opiate substitution therapy can be prescribed by doctors based either at drug treatment centres or in general practice. There are two levels of GPs for OST prescribing. Level 1 GPs can prescribe OST to patients who are already on OST and are continuing therapyand xx patients are allowed per GP at any one time. Level 2 GPs can initiate OST and can prescribe up to xx patients. The aim of the HepLink intervention was to identify and link HCVAb positive patients to tertiary care for treatment of HCV. 14 GPs were involved in the studyand 135 OST patients were enrolled in the study. A nurse liaison was tasked with meeting withpatients, determining HCV Ab status and arranging for follow up tests (RNA or Antigen) andconducting a fibroscan if appropriate (Ab+ and either chronic infection status positive or unknown). Patients who were either chronically infected or Ab+ and awaiting chronic testingwere referred to tertiary care. Demographic data and history of bloodborne virus testing and treatment was collected from patient notes at baseline before the nurse met with patients and at least 6 months afterwards to determine if they had been treated for HCV.

Methods

A Markov model of HCV disease progression was developed to simulate the disease progression of a cohort of individuals that are chronically infected with HCV. The model included Metavir disease stages for liver disease: F0 to F4, decompensated cirrhosis (DC), hepatocellular carcinoma (HCC), liver transplant, post-liver transplant and disease related death (Figure 1). The model was further stratified by whether individuals were infected, on treatment or achieved SVR. Transition probabilities for disease progression and movement between infection, treatment and SVR are given in Table 1. We assume no disease progression or disease related death whilst on treatment, with treatment either leading to SVR (effective cure) or failing and returning back into the infected compartment at the end of 1 year. If SVR is achieved, there is no disease progression from disease stages F0 to F4, reduced disease progression from F4 to HCC and DC, and the same rate of disease progression from DC and HCC onwards. Background mortality is applied to all disease states assuming a male population with mean age of 43 years. We used mortality rates for 5-year age intervals from WHO and converted them to yearly transition probabilities in order to use yearly timesteps.

Supplementary Figure 1

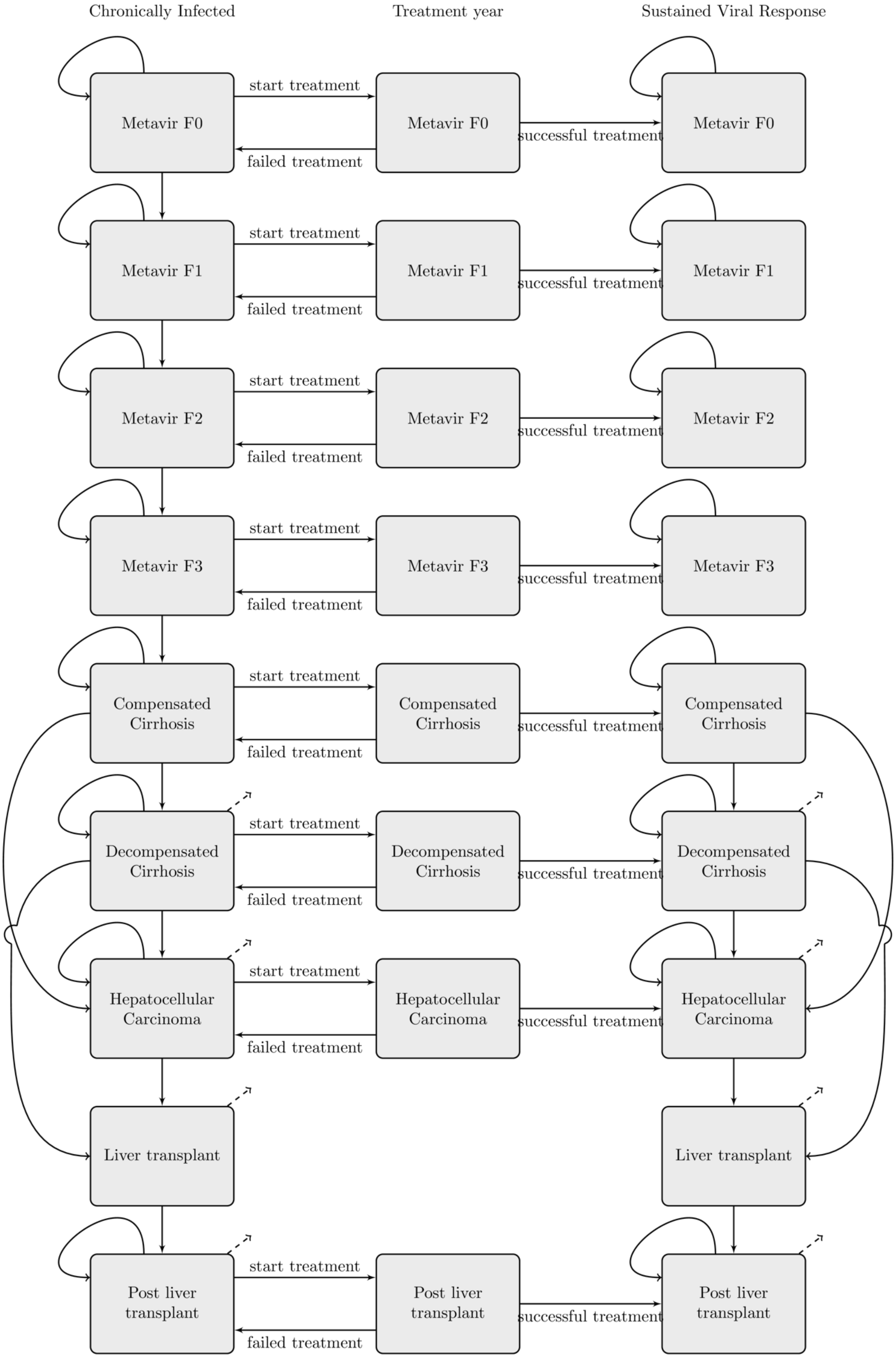

Initial conditions

The study population was modelled as a cohort of chronically infected individuals. As the number of chronically infected individuals in the study population was not fully determined an estimate was used based on the data collected from the HepLink study. There were 100 /135antibody positive patients enrolled in the HepLink intervention (data from baseline collection). Of those who had previously been tested for chronic disease, 81% had a previous RNA+ status (14 attained SVR prior to the start of HepLink). Therefore the initial chronic population size was sampled between 59 and 76 (69-88% chronically infected) and these were then split between Metavir stages F0 to F4 according to the distribution found amongst patients who had a fibroscan score on record. The population of those chronically infected at baseline is used as the initial condition for the Markov model in the comparator. The initial condition for the intervention has the same number of patients altogether, but those who initiated treatment during HepLink were placed in the associated treatment categories rather than chronically infected categories so that all treatment related to HepLink happened in the first year of the modelled time horizon.

Cost and Health Utilities

Costs for treatment of HCV related disease were taken from a recent study (Supplementary Table 2). HepLink intervention costs (in primary care) and HCV treatment costs (in secondary care) were collected as part of the cost-effectiveness study.

Estimation of HepLink intervention costs

Costs for the liaison nurse-led intervention were estimated directly from intervention data using a micro-costing approach from the healthcare system’s (the health and social care system in Ireland) perspective in 2018 Euros. The total costs of the nurse liaison service included intervention set-up and implementation costs. Set up and implementation costs included 4 staff over a 15-month time period and were allocated using a top down approach. Research related costs were identified and excluded from the analysis. A detailed review of the intervention protocol and interviews with key technical staff involved in the planning, implementation and coordination of the intervention were performed to identify all the activities and resources utilized in the integrated model of HCV care that enhances liaison between primary and secondary care in Dublin. The main activities in the intervention included liaison with the general practitioners, patient assessments for HCV status, patient counselling/education, arranging for HCV tests with staff at the GP centre, patient assessment for addiction, fibroscanning patients and referral. Information on the types and quantities of all the resources

utilized for each activity were measured from primary data for each activity, including staff time (liaison nurse, general practitioner, phlebotomist/nurse), and laboratory tests (HCV antibody, HCV antigen, HCV RNA, HCV genotype, fibroscan). Information on the number of patients accessing each service (laboratory investigations and diagnostic tests) and resources used in the pathway was obtained from the study records. The amount of time spent by the liaison nurse, general practitioner, phlebotomist and any other staff involved in the intervention was estimated for each activity using staff time sheets and interviews with the relevant staff. Information on the acquisition costs and replacement values for capital items were gathered from the project's intervention records.

The most up-to-date costs for all the resources used were collected and all retrospective costs were inflated to 2018 Euros using the overall Consumer Price Index for health [38], where necessary. Staff time costs per hour were estimated based on HSE consolidated salary scales [39] information and adjusted for non-pay salary costs in accordance with guidance provided by the Public Spending Code [40]. Unit costs for laboratory investigations (HCV antibody, HCV antigen, HCV RNA, HCV genotype) were obtained from published literature [28] and adjusted to 2018 price levels. Equivalent annual costs for capital items (equipment, set-up costs) were estimated based on the expected service lives using differential rates recommended in the economic evaluation of health technologies guideline for Ireland [37]. The per patient unit cost for fibroscan was estimated from intervention data.

Supplementary Table 1: Intervention costs

Patient related Activities Unit costs	Economic cost per patient	Number of persons	Total Cost
Intervention introduction	€4.76	135	€642.48
GP training-masterclass	€50.54	135	€6,823.06
Patient recruitment	€35.63	135	€4,809.38
Logistical arrangements	€6.17	135	€832.66
Patient assessment for HCV Ab & risk	€26.78	102	€2,731.12
Arranging HCV Ab tests	€7.77	15	€116.59
Patient assessment for HCV RNA	€5.52	77	€424.79
Arranging RNA tests	€7.77	51	€396.40
Counselling	€21.38	102	€2,180.33
Fibroscan	€47.56	43	€2,045.15
Referral	€41.99	28	€1,175.72
HCV antibody test -ve	€49.93	7	€349.51
HCV antibody test +ve	€87.57	14	€1,226.04
HCV HCV-RNA/ NAT test -ve	€94.28	18	€1,697.01
HCV HCV-RNA/ NAT test +ve	€131.92	6	€791.53
			€26,241.78

Estimation of HCV treatment monitoring costs

HCV treatment costs (incurred in secondary care) were estimated based on information gathered through in-depth interviews carried out with the infectious disease clinical nurse specialist at Mater Misericordiae University Hospital where most patients diagnosed with HCV in the HepLink intervention were referred and treated. Based on these interviews and the detailed description of the treatment pathway, we mapped out all the activities, clinical assessments, laboratory investigations and follow up visits performed for an average patient. In short, all patients attending the infectious disease clinic with confirmed chronic infection were considered and prepared for treatment. Patients who drink alcohol excessively are advised to reduce their intake or are linked to relevant support groups and active drug injecting patients are linked with NSP/OST. The treatment of patients follows EASL and Irish guidelines for HCV [28, 41]. Preparation for treatment usually begins with a series of pre-treatment visits (2-3)

where patients are assessed for their medical history and medication use, and a liver ultrasound is done (not necessary in non-cirrhotic patients with fibroscan score <12 kPa,). Patients diagnosed with decompensated liver disease and hepatocellular carcinoma are referred to the hepatology department where they are treated for these complications and, in most cases, for HCV too. We assumed that treatment monitoring costs the same regardless of which department the patient is treated at.

Using the detailed description of the treatment protocol, we identified and quantified the resources used at each stage of the HCV treatment pathway. These resources included HCV DAA medicines, pre-treatment visits (baseline clinical evaluation and laboratory investigations), treatment follow-up visits (treatment monitoring – clinical assessments and laboratory investigations), end of treatment visits, SVR assessment visit and post-treatment follow-up visits. The range of staff involved included clinical (clinical nurse specialist, phlebotomists, intern doctors, senior house officers, consultant physicians, pharmacist) and non-clinical (clerical staff). Most visits were primarily managed by clinical nurse specialists with some involvement of and sign off by physicians.

Staff time costs per hour were estimated based on HSE consolidated salary scales [39] information and adjusted for non-pay salary costs in accordance with guidance provided by the Public Spending Code [40]. Unit costs for laboratory investigations (HCV antibody, HCV antigen, HCV RNA, HCV genotype) were obtained from published literature [28] and adjusted to 2018 price levels. Valuation of the DAA medicines was based on the prices to wholesale (PTW) listed in the Irish Medicines Formulary [42] and adjusted based on guidance for including drug costs in economic evaluations available from the National Centre for Pharmacoeconomics in Ireland [43].

Health utilities for HCV disease progression stages were taken from the literature and a decrement for DAA treatment was applied to 12/52 portion of the base health utility during the treatment year. Once a patient attains SVR the health utility depends on the disease progression stage reached before treatment. For those F0 to F4, the health utility is assumed to increase, whereas for other more severe disease stages the health utility remains the same.

Cost-effectiveness analysis

Using the Markov model, the cohort of patients was followed for 50 years and costs and health utilities attached to each state in the model for each parameter set. The cost-effectiveness analysis is undertaken using a 50 year time horizon and a 5% discount rate for costs and QALYs [37]. The mean incremental cost-effectiveness ratio was calculated (ratio of the mean difference in costs and the mean difference in QALYs between the intervention and the comparator arms) and cost-effectiveness determined using the Irish willingness to pay threshold of €30,000 per QALY [37].

Sensitivity analyses

A probabilistic sensitivity analysis was carried out using 1000 parameter sets sampled from appropriate distributions. The impact of parameter uncertainty on the incremental costs and QALYs was ascertained by an ANCOVA analysis [44]. A cost-effectiveness acceptability curve was obtained to determine the probability of cost-effectiveness at different willingness to pay thresholds.

Supplementary Table 2: Parameters from literature.

Costs			
	**Infection stage cost applies to**	**Mean Yearly Costs (ranges) in Euros**	**Source**
HCV Care Costs			
F0	Infection and treatment	398 (336-482)	Irish HCV medical care costs in the absence of curative treatment [45] Gamma distributions fitted to mean and ranges given, then sampled for probabilistic sensitivity analysis
F1		398 (336-482)	
F2		417 (335-503)	
F3		417 (335-503)	
F4	Infection, treatment and SVR	1790 (990-3164)	
Decompensated cirrhosis		8303 (3945-14637)	
HCC		21992 (15222-29467)	
Liver transplant		137176 (136024-138306)	
Post liver transplant		5337 (4942-5799)	
HCV Treatment Costs			

Treatment monitoring	Treatment	580 +/- 10%	Interview with hospital specialist HCV nursing staff
HCV Drug	Treatment	39730 +/- 10%	Conversion from GBP
All stages monitoring of disease after SVR*	SVR	44 (16-73)	[45] Gamma distributions fitted to mean and ranges given, then sampled for probabilistic sensitivity analysis
Health Utilities			
	Health utility and sampling distribution		Source
F0-F1	0.770 Beta(521.238,155.694)		Health Technology Assessment of HCV treatment [46]
F2-F3	0.659 Beta(168.246,86.672)		
F4	0.552 Beta(47.102,38.538)		
DC (Infected or SVR)	0.447 Beta(123.750,151.250)		
HCC (Infected or SVR)	0.451 Beta(123.750,151.250)		
LT (Infected or SVR)	0.451 Beta(123,750,151.250)		
PLT (Infected or SVR)	0.671 Beta(59.255,29.185)		
Death	0		
F0-F1 SVR	0.8202 Beta(65.868,14.459)		
F2-F3 SVR	0.7193 Beta(58.061,22.579)		
F4 SVR	0.6064 Beta(168.246,86.672)		
Decrement during DAA treatment(applied to 12/52 weeks)	0.06		[47]
Transition probabilties			
F0 to F1	Normal(0.128,0.024)		[34]
F1 to F2	Normal(0.059,0.012)		
F2 to F3	Normal(0.078,0.011)		
F3 to F4	Normal(0.116,0.023)		

F4 to DC	Beta(14.617, 360.173)	[46]
F4 or DC to HCC	Beta(1.933, 136.107)	
DC or HCC to LT	Beta(6.526,210.994)	
PLT to DRD	Beta(22.902,378.883)	
DC to DRD	Beta(147.030,983.970)	
HCC to DRD	Beta(117.103,155.230)	
LT to DRD	Beta(16.276,61.229)	
Standard of care treatment (all states apart from Liver transplant)	Uniform(0.01,0.12)	HepLink Baseline Data
Background mortality	WHO data for Ireland from age 20 onwards converted to yearly transition probabilities	
Other model parameters		
Proportion of treated that achieve SVR if not HCC	Uniform(0.88,0.98)	[35]
Proportion of treated that achieve SVR if have HCC	Uniform(0.7,0.78)	[48]
Relative risk of disease progression from F4 to DC if achieved SVR compared to not	Mean 0.07 (95%CI 0.02-0.3) sampled using lognormal distribution	[36]
Relative risk of disease progression from F4 to HCC if achieved SVR compared to not	Mean 0.23 (95%CI 0.16-0.35) sampled using lognormal distribution	[49]

*In addition to normal disease stage cost for F4 stages onwards

Results

The setup cost was €25,793 and the implementation phase cost €33,405. The direct nurse liaison costs were €26,241 (Supplementary Table 1) Altogether this comes to €85,439. This

cost is applied in year 0 (with no discounting). Supplementary Table 3 shows the total costs and QALYs for the two comparators and the results of the cost-effectiveness analysis.

Supplementary Figure 2: Cost-effectiveness plane showing results of the probabilistic sensitivity analysis

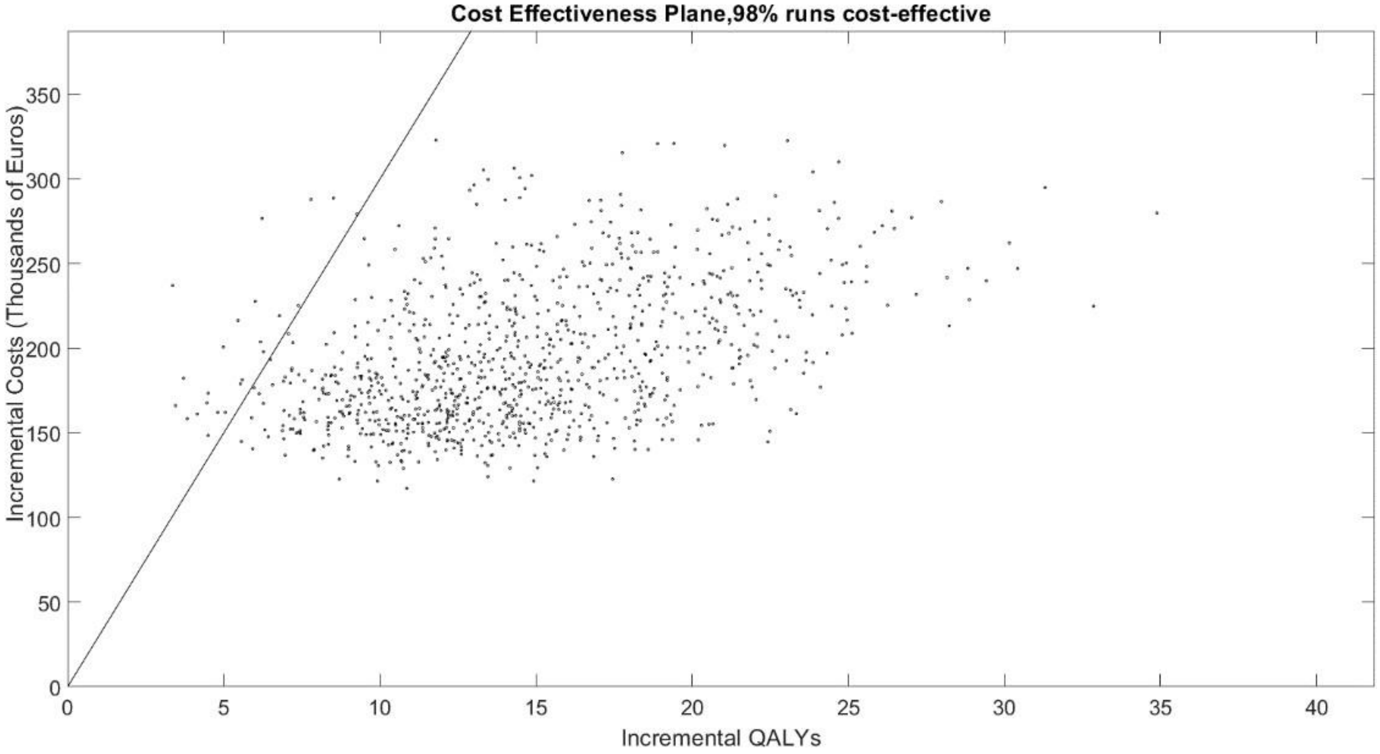

Supplementary Table 3: Cost-effectiveness results

Scenario	Costs	Incremental Costs	QALYs	Incremental QALYs	ICER € per QALY
Comparator	€2,522,936		666		
Intervention	€2,717,718	€194,782	681	15	€13,255

Supplementary Figure 3: Cost-effectiveness acceptability curve showing the probability the intervention is cost-effective at different willingness to pay thresholds

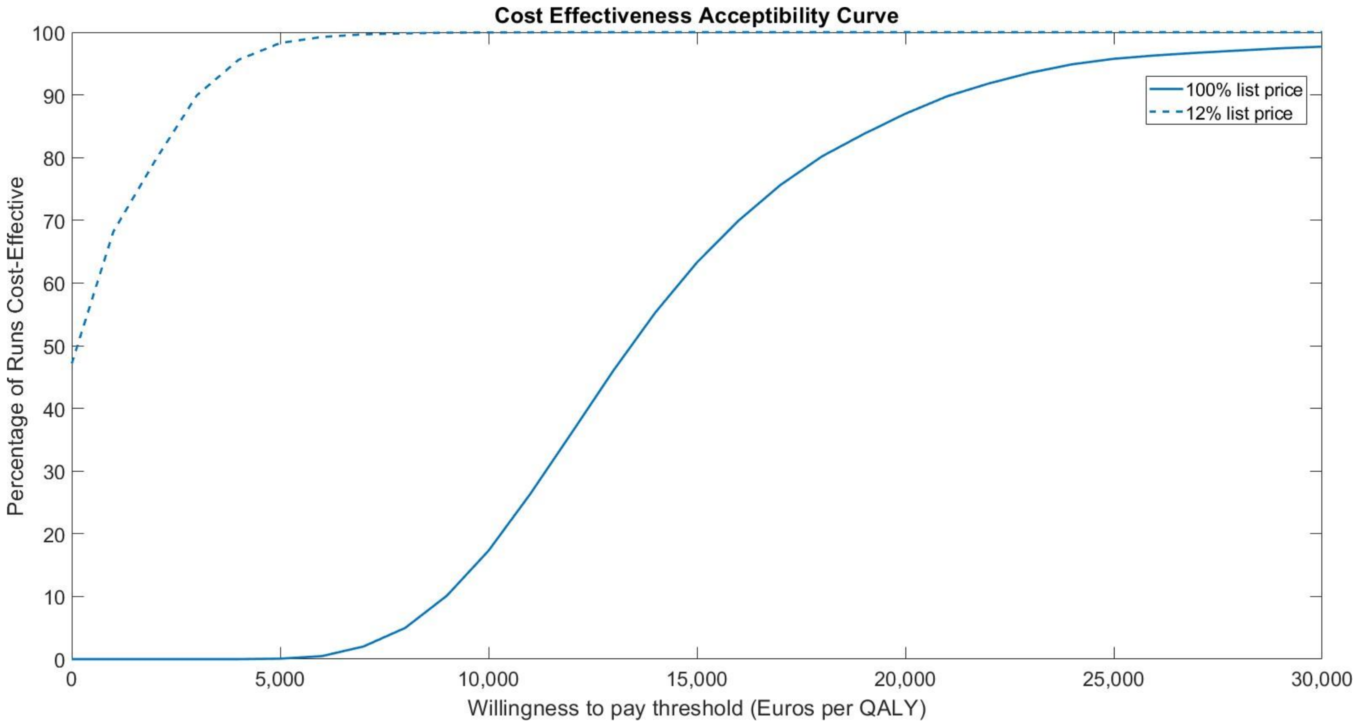

Supplement: Multimedia Appendix 1 [file ijmr_v11i2e35300_app1.pdf]
